# Urinary profiles of progestin and androgen metabolites in female polar bears during parturient and non-parturient cycles

**DOI:** 10.1093/conphys/cox023

**Published:** 2017-04-24

**Authors:** Katrina K. Knott, Gabriela F. Mastromonaco, Megan A. Owen, Andrew J. Kouba

**Affiliations:** 1 Conservation and Research Department, Memphis Zoo, 2000 Prentiss Place, Memphis, TN 38112,USA; 2 Toronto Zoo, 361A Old Finch Avenue, Scarborough, ON, M1B 5K7, Canada; 3 Institute for Conservation Research, San Diego Zoo Global, 15600 San Pasqual Valley Road, Escondido, CA 92027,USA; 4 Department of Wildlife, Fisheries and Aquaculture, A205 Thompson Hall, Box 9690, Mississippi State University, MS 39762,USA

**Keywords:** embryonic diapause, induced ovulation, non-invasive, reproduction, urine, ursid

## Abstract

Due to the environmental and anthropogenic impacts that continue to threaten the reproductive success of polar bears, a more detailed understanding of their reproductive cycle is needed. Captive populations of polar bears provide an excellent opportunity to learn more about the reproductive physiology of the species. Progestin (P4) and androgen (T) metabolites in urine, and their ratio (P4:T), were examined during 11 reproductive cycles of captive female polar bears (*n* = 4) to characterize the steroid hormone profile during pregnancy and determine possible variations related to reproductive failure. The concentration of hormone metabolites in urine were determined through enzyme immunoassay. Reproductive cycles were classified as pregnant (*n* = 3), anovulatory (*n* = 4) and ovulatory-non-parturient (*n* = 4) based on the changes in urinary hormone metabolite values and cub production. In the absence of a lactational suppression of estrus, elevated androgen concentrations suggested resumption of follicular development within 3 weeks of parturition. Breeding behaviours were most often observed when androgen values were at their highest or in decline. Ovulation was identified by a return to basal androgen concentration and elevation of progestins within 1–4 weeks after breeding. As a result, urinary concentrations of progestins were greater than androgens (P4:T ratio ≥ 1.0) during ovulatory cycles whereas the P4:T ratio was <1.0 when females were anovulatory. Progestins and the P4:T ratio of parturient cycles were greatest beginning in June/July (17–20 weeks after breeding) and reached a peak at 24–37 weeks (mid-October/mid-November, 4–9 weeks before birth of cubs). Non-invasive monitoring of hormone metabolites in urine provided a rapid determination of endocrine function for improved husbandry and reproductive management of polar bears in captivity. Further research is warranted to understand the reproductive endocrinology of polar bears and its impact on conservation and management of this species in captivity and the wild.

## Teaser

Management of polar bears is improved by understanding the annual changes in progestins and androgens that regulate key reproductive events. Physiological data gathered from captive polar bears provide insight into potential causes of reproductive failures in wild polar bears threatened by a changing Arctic.

## Introduction

Polar bears (*Ursus maritimus*) are adapted to the Arctic climate through physiological modifications such as seasonal breeding, delayed implantation, fasting during maternal denning and extended periods of lactation (Palmer *et al.*, 1988; Derocher *et al.*, 1992; Amstrup, 2003). The breeding period for free-ranging polar bears is concentrated between March and June (Palmer *et al.*, 1988; Stirling and Ortsland, 1995; Rosing-Asvid *et al.*, 2002; Spady *et al.*, 2007). Following successful breeding, free-ranging female polar bears enter maternal dens in the fall where they give birth and nurse cubs while undergoing a period of fasting (Watts and Hansen, 1987; Ramsay and Stirling, 1988; Atkinson and Ramsay, 1995; Ferguson *et al.*, 2000). A 33% mortality rate due to pregnancy loss or neonatal mortality has been estimated for free-ranging polar bears (Derocher *et al.*, 1992). Reproductive failures are anticipated to increase as a direct and indirect response to warming temperatures and changes in the Arctic sea-ice habitat (Atkinson and Ramsay, 1995; Derocher *et al.*, 2004; Cherry *et al.*, 2009; Wiig *et al.*, 2015; Lunn *et al.*, 2016). Changes in body condition, disease, contaminants and reproductive success (and their interaction) have been ranked as important measures of health for polar bears (Bowen *et al.*, 2015; Patyk *et al.*, 2015; Atwood *et al.*, 2016).

The reproductive cycle of polar bears has been described to be largely controlled by changes in photoperiod (i.e. short–long day switch, <10-h day length threshold and rate of day length change), which determines the breeding, implantation and consequently parturition date in ursids (Spady *et al.*, 2007). During breeding, androgens are converted to estrogens via aromatase to stimulate follicular development (Norris, 1980; Staub and DeBeer, 1997). Androgens and estrogen also have a synergistic action on the pre-ovulatory surge of luteinizing hormone (LH) by the anterior pituitary that signals ovulation and consequent production of progestins by the corpus luteum (CL) during the luteal phase (Nubbemeyer, 1999; Bakker and Baum, 2000; Senger, 2005). Elevated progestins in fall and winter have been used to identify pregnancy in polar bears; however, females also experience reproductive cycles during which progestins are elevated and do not birth cubs (Derocher *et al.*, 1992). It is unknown whether this lack of cub production is a result of failure to conceive or indicates pregnancy failure during embryonic diapause. Hormonal changes required for implantation and resumption of embryo development following embryonic diapause have been well described in black bears (*Ursus americanus*) and giant pandas (*Ailuropoda melanoleuca*) (Tsubota *et al.*, 1998; Steinman *et al.*, 2006). Similar hormonal events are expected to occur in polar bears, but the sequence of these events have not been completely defined.

Serially collected data gathered from captive populations provide a valuable reference of physiological measures that can be used for comparison to single-point data observations in free-ranging individuals. The collection of physiological data from captive populations can allow researchers to conduct more thorough investigations into animal physiology that cannot be performed under standard field conditions. For example, collection of longitudinal hormonal data from free-ranging polar bears is not possible due to logistical constraints and the challenges of capturing and immobilizing the same individual several times over the duration of their year-long reproductive cycle. Concentrations of hormones in free-ranging polar bears are often reported from a single time point (e.g. cross-sectional studies), which does not allow for an evaluation of the natural fluctuations in hormones in the same individual over time with changes in physiological status or season (e.g. longitudinal studies). As a consequence, studies of the potential negative impacts of nutritional stress or environmental contaminants on hormones in free-ranging polar bears have been limited by the lack of information regarding the natural annual variation of hormones throughout the duration of a females’ year-long reproductive cycle, and potential differences among cohorts (e.g. females with and without cubs; pregnant and non-pregnant females; Haave *et al.*, 2003; Knott *et al.*, 2011; Oskam *et al.*, 2011; Gustavson *et al.*, 2015).

Reproductive management of captive polar bears also can be challenging due to limited information on when to introduce animals for breeding, husbandry demands, space constraints and staff requirements to avoid animal aggression. For example, care of pregnant females includes preparation of maternity dens, supply of appropriate nesting materials and separation from males and other conspecifics. A useful conservation tool for research and management of many threatened species is non-invasive reproductive monitoring of gonadal and placental function by hormone analyses (Kersey *et al.*, 2010; Knott *et al.*, 2013; Schwarzenberger and Brown, 2013; Kersey and Dehnhard, 2014). Previously, Stoops *et al.* (2012) reported that changes in progestin and androgen metabolites could be measured in polar bear faeces using an enzyme immunoassay (EIA) and the results were successful for determining late term pregnancy. Endocrine analyses of faeces, however, are labour intensive due to the laboratory preparation prior to analyses such as faecal drying and hormone solvent extraction, and requires ample storage capacity that is not possible at all zoo facilities. As a result, a long-term monitoring program for captive management using faecal analyses is difficult to maintain. In contrast, the analysis of hormone metabolites in urine avoids many of these challenges and provides a rapid determination of endocrine function for management guidance in near real-time (within hours of obtaining samples). We hypothesized that, similar to other ursid species, hormone metabolites could be quantified in female polar bear urine, provide information on the stage of the reproductive cycle, and determine pregnancy.

To test our hypotheses, urine was collected from polar bears at three North American zoos and metabolite concentrations of urinary progestins (progesterone, P4), androgens (testosterone, T) and the P4:T ratio were evaluated. Our objectives were to: (i) determine whether progestin and androgen EIAs for evaluating urinary hormone metabolites would work in polar bears; (ii) quantify and profile ovulatory and anovulatory cycles from urine collections; and (iii) compare the urinary hormone profiles of ovulatory cycles that resulted in parturient and non-parturient outcomes. These data are anticipated to guide husbandry and management decisions for polar bears held in captivity and allow animal managers to plan breeding pair introductions, support term pregnancy and increase neonatal survival. Greater knowledge of the reproductive physiology of polar bears will also provide further information regarding the timing and plasticity of reproductive events and insight into potential causes of reproductive failures.

## Materials and methods

Female polar bears were housed at the Toronto Zoo (TZ: female, Studbook (SB)1132, date of birth (DOB) 2000; female, SB1131, DOB 2000), Memphis Zoo (MZ: SB1147, DOB 2002) and San Diego Zoo (SDZ: SB1041, DOB 1995). Females were selected based on those institutions that were able of collect urine samples during all months of the year. At TZ and MZ, the male was slowly introduced to the female for breeding access after staff determined that the female exhibited affiliative interest towards the male through enclosure barriers (AZA Bear TAG, 2009). The male and female at SDZ remained on exhibit together year-round unless behavior indicated a preference for self-sequestration. Courtship behaviours were allowed to occur naturally according to animal interest and without interference. Dates that the male mounted the female were recorded by animal care staff at each institution. Although copulations appeared successful, it could not be determined whether males ejaculated during couplings. Males were separated from the female when managers determined that the female was no longer interested in mating advances. Females were isolated from conspecifics in a separate enclosure 2–3 months before anticipated parturition. Ages of females ranged 9–18 years during the study period and thus all were considered to be reproductively mature. Female SB1041 was given progestin injected contraceptives (Depo-Provera^®^) during 2004–05, and implanted with a subdermal contraceptive [gonadotropin releasing hormone agonist; deslorelin (Suprelorin^®^ implant)] during December 2006. This female was considered to have resumed cycling during the time of this study (2011–13) as breeding was observed each year.

### Sample collection and processing

A calendar year for each female was considered to be 1 reproductive cycle. Urine was collected over the course of 3 parturient cycles for SB1132 (2011–13; 70–146 samples/year). Cubs were removed from the female shortly after birth to improve neonatal survival, and to allow the female to breed the following spring. Sampling collection during 2011 was interrupted by changes to exhibits, thus samples were not collected during July and Aug. The remaining females were non-parturient (SB1131, *n* = 2 cycles, 2012–13; SB1147, *n* = 4 cycles, 2011–14; SB1041, *n* = 2 cycles, 2011–12; 90–154 samples/cycle/year). Urine samples were collected from indoor animal enclosures shortly after they were voided. Urine was collected with a syringe, taking care to avoid debris. Urine was transferred into labelled tubes and stored at −20°C until endocrine analysis.

### Endocrine assays

Detection of progestin and androgen metabolites in urine were performed using a single antibody competitive EIA as previously described by this laboratory (Willis *et al.*, 2011; Knott *et al.*, 2013; Gosinski *et al.*, in review). Antibodies (progesterone, P4, CL425; testosterone, T, R156/7) and hormone–horseradish peroxidase (HRP) conjugates were provided by C. Munro, Clinical Endocrinology Laboratory, University of California Davis. The P4 assay employed a rabbit anti-4-progesterone-11-ol-3,20-dione–BSA monoclonal antibody (1:6000) and progesterone-3CMO-HRP conjugate (1:60 000) as described previously by Graham *et al.* (2001). The P4 antibody cross-reacts with 100% progesterone (4-pregnen-3,20-dione), 188% 4-pregnen-3α-ol-20-one, 172% 4-pregnen-3β-ol-20-one, 147% 4-pregnen-11α-ol-3,20-dione, 94% 5α-pregnen-11-3β-ol-20-one, 64% 5α-pregnan-3α-ol-20-one, 55% 5α-pregnane-3,20-dione, 12.5% 5β-pregnane-3β-ol-20-one and <10% for all other tested steroids. Total urinary androgen metabolites were detected using a polyclonal rabbit anti-testosterone antibody (1:25 000) and testosterone-3CMO-HRP conjugate (1:25 000). The testosterone antibody cross-reacted with 100% testosterone, 28% 5 α-dihydrotestosterone, 0.78% androstenedione, 0.19% androsterone, 0.05% dehydroepiandrosterone, 0.03% progesterone and <0.01% for estradiol-18 β, pregnenolone and cortisol (Kersey *et al.*, 2010; C. Munro, pers. comm.). Standards for the P4 (4-pregnen-3,20-dione, Sigma-Aldrich, St. Louis, MO, USA) assay ranged 5.0–10 000.0 pg/mL, and the T standards (testosterone, Sigma-Aldrich, St. Louis, MO, USA) ranged 23.0–24 000.0 pg/mL. Standards and samples were assayed in triplicate, and intra-assay variation was less than 10%. Serially-diluted urine samples exhibited parallelism to the standard curve (*r* = 0.994 and 0.987). Polystyrene microtiter plates were coated with antibody and stored overnight at 4°C. Urine samples and hormone-HRP were applied to compete for binding sites on the antibodies. Azino-bis-3-ethyl benzthiazoline-6-sulfonic acid (ABTS) was used as the substrate and hydrogen peroxide as the catalyst to detect the percent of hormone specific-HRP conjugate bound to the antibody using an MRX Revelation plate reader (Thermo Scientific, Rochester, NY, USA). The concentrations of endocrine metabolites were determined by the inverse of the bound fraction as compared to a standard curve. Because urinary hormone concentrations were low, the majority of samples were run neat or at 1:2 dilution. To avoid errors due to variations in sample collection, a cut-off value of 0.1 ng hormone/ml urine was used as a minimum detection limit. Urine samples having hormone values below this limit for either assay were excluded from further analysis (<10% of samples for each reproductive cycle).

The progesterone and testosterone assays were chosen due their common use in zoo research laboratories, the more rapid turn-around time for results, and similar or improved detections of hormone metabolites over other assays examined. Urinary progestin concentrations using the antibodies against pregnanediol-glucuronide (R13904) resulted in concentrations below the detection limits of the assay as performed in our laboratory. Pretreatment of urine samples by enzyme hydrolysis (EH) with enzyme β-glucuronidase/arylsulfatase (Roche Diagnostics, obtained from *Helix pomatia*) using procedures adapted from Al-Dujaili (2006) and Steinman *et al.* (2013) were explored. Final testosterone concentrations of EH treated versus non-EH treated samples were 4-fold greater when urinary concentrations were considered to be at baseline, and up to 20-fold when testosterone values were most elevated during the breeding season. Reproductive patterns, however, remained the same for both EH and non-EH treated samples (*r* = 0.719, *P* < 0.001). Unlike the testosterone results, EH did not improve detection of progestins in urine from polar bears, and resulted in a reduction of final progestin concentrations below the detection limits of the assay. These pretreatment methods also added another step that lasted overnight, which was impractical for rapid monitoring.

### Creatinine assays

The concentration of urinary creatinine was used to account for variations in water content between samples as performed in other studies (Kersey *et al.*, 2010; Willis *et al.*, 2011). In brief, creatinine was evaluated by placing 50 μl each of H_2_O, 0.75 N NaOH and 50 μl of 0.4 N picric acid added to 1:20 diluted urine sample. Creatinine concentrations were determined by comparison of optical density to a standard curve ranging from 6.25 to 100 μg/ml (Creatinine Solution; Arbor Assays, Ann Arbor, MI, USA). Hormone data from samples having creatinine concentrations <0.2 mg/ml were excluded from further analyses. Hormone concentrations were reported as ng hormone/mg creatinine.

### Data analyses

Hormone profiles were aligned to the last day of observed breeding (Day 0). Urinary androgens were elevated before and during observed breeding events consistent with the approximate time of follicular development. For each year-long cycle, the mean of androgen values from the first week after observed breeding (Week 1) to the end of the cycle year (Week 33) was considered the post-breeding androgen baseline [combined mean ± standard deviation (SD), 0.4 ± 0.3]. Androgen values 2 SD above the post-breeding mean were considered to be elevated. The mean progestin value for all samples collected 10 days before the last day of observed breeding activity (Day 0) was considered to be the progestin baseline (combined mean ± SD, 0.3 ± 0.1). Progestins were considered to be elevated and indicative of ovulation when the progestin concentration was 2 SD above that of baseline concentrations for 2 consecutive sampling days (Kersey *et al.*, 2010). The length of the pregnant luteal phase was determined by the number of days between the date that progestins were elevated to parturition.

Reproductive cycles that concluded with the birth of cubs were considered parturient. Cycles were classified as ovulatory-non-parturient if progestins were elevated after breeding, but no cubs were born. Anovulatory cycles were defined as calendar years when the progestin concentrations of the female were not elevated after breeding. Multiple years were pooled by female for each reproductive category and data visualized as weekly means from breeding. As elevated progestins provide a negative feedback on the production and release of estrogens and androgens during pregnancy (Senger, 2005), the ratio of progestins to androgens (P4:T ratio) was also calculated for improved visualization of reproductive cycles. The average of P4:T ratios each week were also aligned to the last day of observed breeding to compare annual patterns among reproductive categories.

The Shapiro Wilks test determined that hormone concentrations were not normally distributed (*P* < 0.05). Therefore, a Kruskal–Wallis One Way Analysis of Variance on Ranks with Dunn’s test for multiple comparisons was used to compare hormone concentrations between reproductive categories and stages of the reproductive cycle. Microsoft Excel (Microsoft Office Systems) was used for data organization and iterations. Sigma Plot 12.0 (Systat Software Inc., San Jose, CA, USA) was used for graphical representation and statistical analyses. A *P*-value of <0.05 was used to determine significance. Data are expressed as the mean ± standard error of the mean (SEM) unless otherwise stated.

## Results

### Breeding activity and urinary androgens

Observed breeding activity for all females occurred during late January through the end of May (Table 1). The parturient female bred between January 30 and March 23, and the duration of breeding attempts occurred over 4–20 days. Breeding activities occurred over a greater number of months for females with anovulatory cycles (5 months) compared to parturient cycles (3 months). Females having ovulatory-non-parturient cycles bred for a duration of 6–17 days similar to that of the parturient female. During 3 of the 4 ovulatory-non-parturient cycles examined, breeding occurred during February 16 to March 23. Breeding occurred in April though early May for the other ovulatory-non-parturient cycle.

**Table 1:** Dates of observed breeding for pregnant, ovulatory and anovulatory female polar bears held at the Toronto Zoo [studbook number (SB) 1132 and SB1131], Memphis Zoo (SB1147) and San Diego Zoo (SB1041). The date of parturition for SB1132 occurred on October 11, December 6 and November 10 for pregnancies occurring in 2011, 2012 and 2013, respectively

	Dates of breeding activity
Pregnant	
SB1132 (2011)	March 3–23
SB1132 (2012)	January 30–February 3
SB1132 (2013)	February 23—March 8
Ovulatory-non-parturient	
SB1147 (2013)	March 17–23
SB1147 (2014)	February 16–23
SB1041 (2011)	February 14–24
SB1041 (2012)	April 21–May 8
Anovulatory	
SB1147 (2011)	February 26–30; April 2–7; May 28–30
SB1147 (2012)	January 24–February 2; March 15–26
SB1131 (2012)	March 3–8; April 21–29
SB1131 (2013)	March 29–April 7

**Figure 1:**
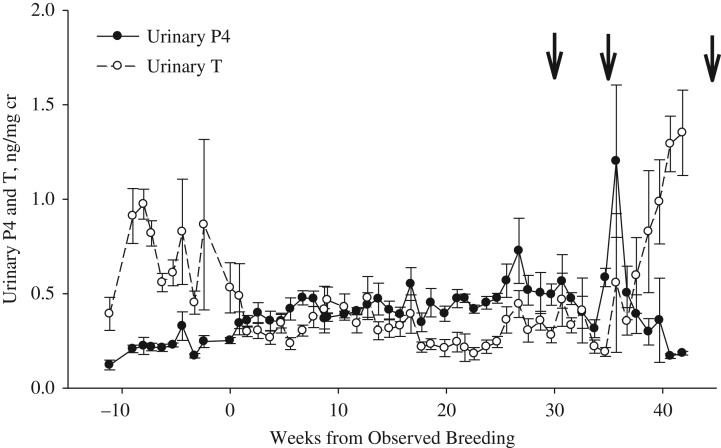
Weekly progestin (P4) and androgen (T) concentrations (mean ± SEM) in urine from a pregnant captive polar bear (SB 1132; *n* = 3 reproductive cycles; 2011–13) as aligned to the last day of observed breeding. The arrow represents the date when cubs were born each year.

**Figure 2:**
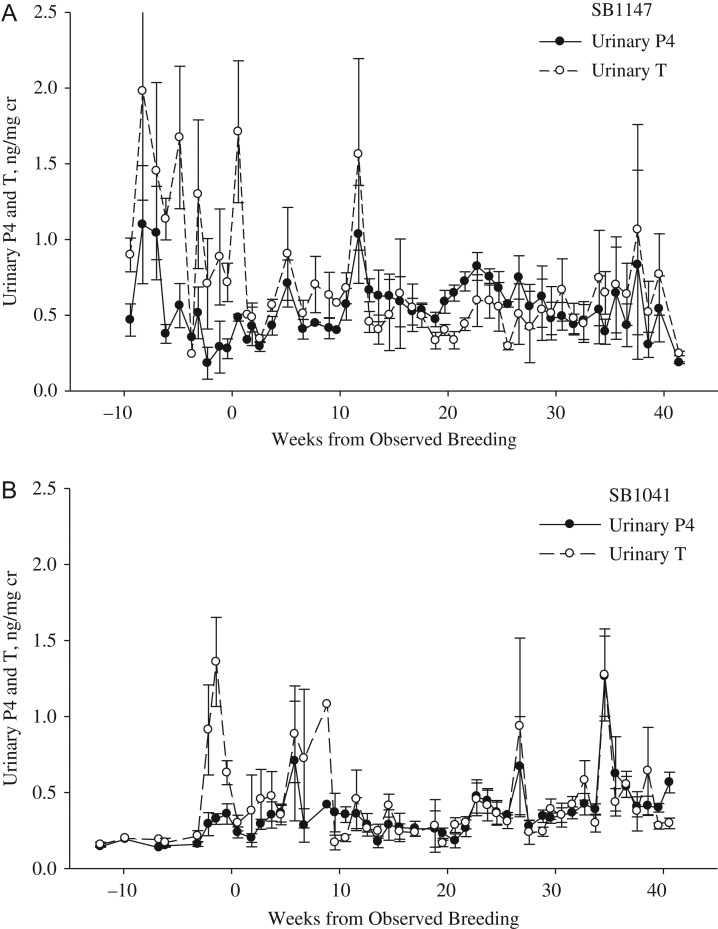
Weekly progestin (P4) and androgen (T) concentrations (mean ± SEM) in urine from captive female polar bears during presumed ovulatory-non-parturient cycles as aligned to the last day of observed breeding (*n* = 2 reproductive cycles for each female). (**A**) The cycles for SB1147, 2013–14 and (**B**) the cylces for SB1041, 2011–12.

**Figure 3:**
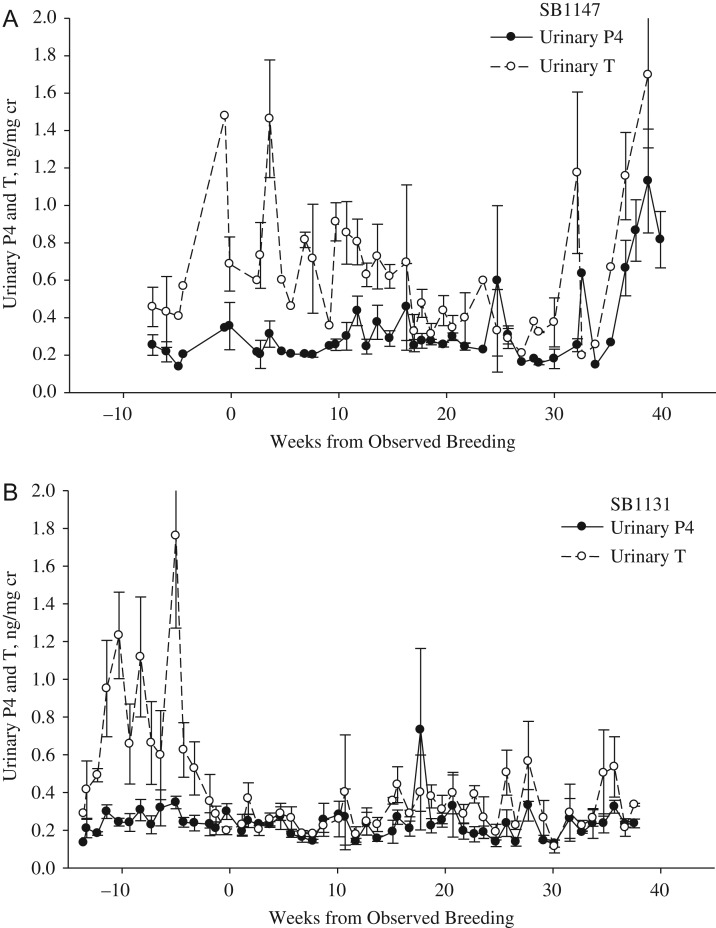
Weekly progestin (P4) and androgen (T) concentrations (mean ± SEM) in urine from captive female polar bears during presumed anovulatory cycles as aligned to the last day of observed breeding (*n*= 2 reproductive cycles for each female). (**A**) The cylces for SB1147, 2011–12 and (**B**) the cycles for SB1131, 2012–13.

**Figure 4:**
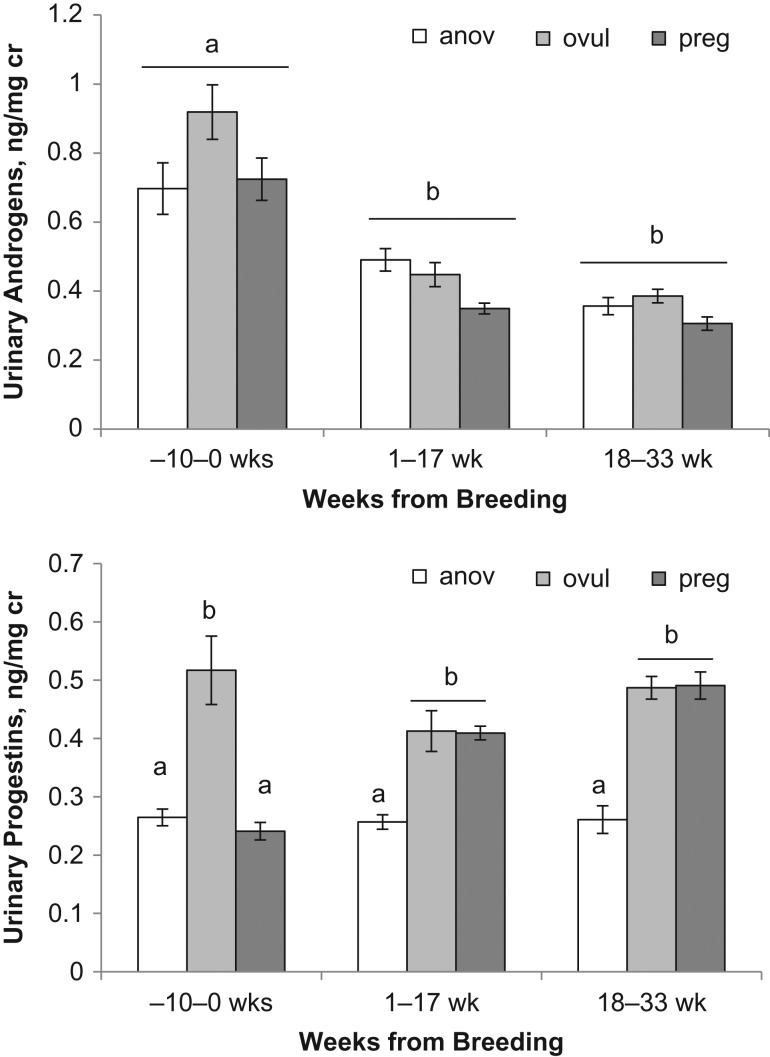
Urinary progestin and androgen concentrations (mean ± SEM) of captive female polar bears during the presumed period of embryonic diapause (1–17 weeks) and the secondary surge in progestins (18–33 weeks) for pregnant cycles (*n* = 3) in comparison to the same time interval for ovulatory-non-parturient (*n* = 4) and anovulatory (*n* = 4) cycles. Different letters indicate significant differences between reproductive categories and time interval.

For all cycles, urinary androgen concentrations were significantly higher (*P* < 0.001) during Week −10 to Week 0 of last breeding date (0.8 ± 0.1 ng/mg cr) than androgen values post-breeding (Weeks 1–33, 0.4 ± 0. 1 mg/mg cr; Figs 1–4). For pregnant cycles, an increase in urinary androgens above the post-breeding baseline occurred 18–22 days after the cubs were born and removed from the mother. Androgens remained elevated above post-breeding baseline values for 50–97 days for pregnant cycles (Fig. 1) and 12–120 days for ovulatory-non-parturient cycles (Fig. 2). For all ovulatory cycles (pregnant and non-parturient), androgens were in decline during the period of breeding activity and at post-breeding concentrations within 2 weeks of mating (ranged 0–14 days; Figs 1 and 2). Anovulatory cycles for female SB1131 also exhibited an androgen decline in relation to breeding activity, while androgen values remained elevated well into the summer for the anovulatory cycles for female SB1147 (Fig. 3).

### Parturient luteal phase

During pregnant cycles, an elevation of progestins occurred within 7–28 days from the last observed breeding (Fig. 1). Elevated progestins remained 1.8-fold greater than basal progestins and 1.4-fold above androgens for 17 weeks post-breeding (~119 days). A secondary increase in progestins (2.2-fold above basal progestins) began at ~17–20 weeks from breeding, and lasted for a duration of 83–188 days before parturition. During the two pregnancies in which all months were sampled, the highest concentrations of urinary progestins occurred at 27 and 36 weeks (October and November). Thereafter, progestins declined while androgens began to increase. This pattern in hormonal activity occurred for ~60 days before parturition. Parturition occurred 202–307 days (29–44 weeks) after breeding.

### Non-parturient luteal phase

Progestin concentrations during the follicular phase of ovulatory-non-parturient cycles were greater (*P* < 0.001) than those for pregnant and anovulatory cycles (Fig. 4). Time of ovulation and the beginning of the luteal phase could be identified within 2–4 weeks of the last breeding date for 3 of 4 ovulatory-non-parturient cycles (SB1147 2014, SB1041 2011, SB1041 2012). An increase above follicular level progestins during cycle SB1147 in 2013 occurred 83 days after last observed breeding date. Because this progestin increase occurred in June, it is likely that this surge was actually a secondary progestin increase and that ovulation occurred earlier than could be estimated from last breeding observation. Although daily progestin concentrations of ovulatory-non-parturient cycles were highly variable (Fig. 2), the overall mean progestin concentrations of ovulatory-non-parturient cycles during Weeks 1–17 and Weeks 18–33 post-breeding were not statistically different from overall luteal progestin concentrations during pregnancy (Fig. 4). In contrast, concentrations of progestins after breeding for anovulatory cycles were significantly lower (*P* < 0.001) than those of ovulatory-parturient and non-parturient cycles (Fig. 4).

The mean concentration of post-breeding androgens were not significantly different (*P* > 0.05) among pregnant (0.3), ovulatory-non-parturient (0.4) and anovulatory cycles (0.4) (Fig. 4). However, daily androgen values post-breeding were more variable during non-parturient cycles (anovulatory, SD = 0.32; ovulatory-non-parturient, SD = 0.29) compared the androgen concentrations observed during pregnancy (SD = 0.18).

### Identifying reproductive status through evaluation of the P4:T ratio

**Figure 5:**
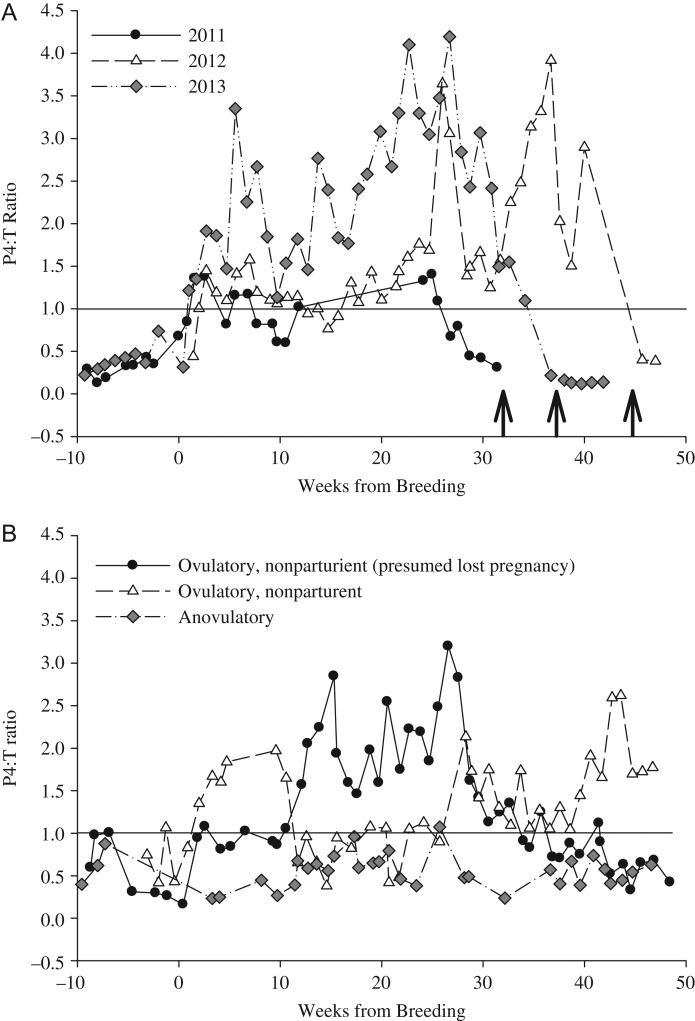
The ratio of progestins to androgens (P4:T ratio) in urine from captive female polar bears during (**A**) 3 pregnant cycles (SB1132; 2011–13) and (**B**) during 3 non-parturient cycles (● SB 1147, 2014; ∆ SB 1147, 2011; ♦ SB 1101, 2011). Data are shown as weekly average from the last day of observed breeding. The arrows in (A) represent the dates when cubs were born each year.

During all 3 pregnant cycles, the P4:T ratio was at or greater than 1.0 within 1–2 weeks of breeding. In two pregnancies, the P4:T ratio varied from 0.5 to 1.5 during the period of embryonic diapause (after breeding through 17–20 weeks) whereas the P4:T ratio during the third pregnancy ranged from 1.5 to 3.5. The highest P4:T ratio during the 2012 and 2013 pregnancies occurred between 24 and 37 weeks (mid-October–mid-November, 7–9 weeks before birth of cubs; Fig. 5A). The highest P4:T ratio during the 2011 pregnancy occurred at 24 weeks post-breeding and cubs were born 4 weeks after this peak. The shorter interval between peak P4:T ratio and parturition may be due to sampling error, as there was a lack of urine samples available from this female during the latter stages of the 2011 pregnancy.

Progestin concentrations for ovulatory-non-parturient cycles were elevated above androgens for a similar proportion of time as for pregnant females (SB1041, 54% and SB1147, 77 versus 72% of the pregnant cycle). As a result, the P4:T ratio during reproductive cycles of ovulatory-non-parturient females were similar to the pregnant cycle in that the ratio was at or greater than 1.0 within 2 weeks of breeding. This pattern of post-breeding P4:T ratio suggests that pregnancy failure likely occurred during the period of embryonic diapause or during the gestational period. In contrast, urinary progestin concentrations were similar or below androgens throughout the duration of anovulatory cycles (Figs 3 and 4), and therefore resulted in a P4:T ratio that remained below 1.0 throughout the reproductive cycle (Fig. 5B).

## Discussion

This study revealed that the stages of the reproductive cycle and pregnancy status of polar bears could be characterized using a non-invasive hormone assay technique to quantify urinary progestin and androgen metabolites. The data revealed distinct hormone profiles that identified ovulation, and assisted in the determination of parturient and non-parturient outcomes. Breeding behaviours were most often observed when androgen values were at their highest or in decline. Ovulation was identified by the elevation of the P4:T ratio above 1.0 within 2 weeks of breeding that occurred as result of both the increase in progestins and the corresponding decrease in androgens. Pregnancy detection in polar bears was most notable by the continued elevation of progestin concentrations above those of androgens for the majority of the reproductive cycle. This hormonal pattern and the duration of pregnancy from breeding to the birth of cubs in this study (202–307 days) was similar to those described in previous studies (range: 164–294 days; Tumanov, 2001; Stoops *et al.*, 2012). The time interval between mating and the increase in urinary progestins signalling ovulation may be a result of the delay in the production of progestins from the CL and urinary excretion that could be detected by the EIA. However, ovulation could also be delayed for several days following copulation. Based off of CL measurements from free-ranging Greenland polar bears, Rosing-Avid *et al.* (2002) expected that mating behaviours occurred as early as one month before estimated ovulation.

The pattern and increase of urinary androgen metabolites corresponded well with behavioural estrus and the time of female sexual receptivity in polar bears as has been described for other mating induced ovulators (Staub and DeBeer, 1997; Nubbemeyer, 1999; Bakker and Baum, 2000). Breeding events in this study ranged from January to May as reported previously for captive females (Tumanov, 2001; Meyerson and Long, 2010; Stoops *et al.*, 2012). Within 3 weeks of removal of cubs, elevated androgen concentrations suggested resumption of follicular development and may be considered the minimal interval for polar bears in absence of lactational suppression. Stirling *et al.* (2016) also reported that a pair of wild polar bears mated within a week of the female separating from her cub. Because the reproductive cycle of polar bears occurs over a full calendar year, a rapid return to estrus is likely an adaptive strategy that allows females to be available for breeding in late winter and spring when androgens in males are at their highest (Palmer *et al.*, 1988; Spady *et al.*, 2007; Curry *et al.*, 2012). Successful mating that induced ovulation in this study occurred between late January and March, which is earlier than the mating time reported for polar bears breeding in the wild. Managers of captive polar bears should consider this early estrus period, and allow males access to females as early as January for females that have yet to become pregnant, or in cases of early cub removal and neonatal mortality. Males with access to females only after April–May could miss opportunities to mate with females that entered estrus earlier in the year. Furthermore, extended access of males to females after breeding may be stressful for some females as they enter into the period of embryonic diapause.

The period of embryonic diapause in this study (~4 months) is in alignment with previous reports for polar bears and black bears (~5 and 3 months, respectively; Derocher *et al.*, 1992; Tsubota *et al.*, 1998). The CL during embryonic diapause is thought to be largely nonfunctional, and the elevated progestins detected in fall have been related to a 2–4.5-fold increase in luteal volume that occurs before and during implantation (Wimsatt, 1963). In the present study, a secondary increase in progestins began during June/July, which was 17–20 weeks after breeding. Progestins remained elevated for a duration of 83–188 days before the birth of cubs which was similar to previous reports of polar bears, brown bears (*Ursus arctos*) and black bears (Tsubota *et al.*, 1987, 1998; Palmer *et al.*, 1988; Hellgren *et al.*, 1990; Sandell, 1990; Spady *et al.*, 2007). The highest concentration of progestins (and the highest P4:T ratio) occurred at 24–37 weeks (mid-October–mid-November, 4–9 weeks before birth of cubs) corresponding to the approximate time of embryo implantation. Ursid cubs grow quickly after implantation as was determined through the visualization of a foetus in brown bears and giant pandas at 30 days before parturition that measured only 2 cm (Tsubota *et al.*, 1987; Sutherland-Smith *et al.*, 2004). Foetal development has yet to be recorded by ultrasound in polar bears.

The depression of androgens after breeding and ovulation is consistent with the negative feedback mechanism following the production of elevated progestins in support of placental and foetal development (Senger, 2005). The suppression of androgens during embryonic diapause was most evident during the pregnant cycle when examining the P4:T ratio. Tsubota *et al.* (2001) similarly described that the CL was capable of producing only minimal concentrations of androgens or estrogens during delayed implantation in black bears. Contrary to the pregnant luteal phase, the luteal phase of ovulatory-non-parturient cycles was characterized by greater daily fluctuations in androgens and progestins farther into the summer. Polar bears are considered to be polyestrus seasonal breeders and can enter into at least two periods of estrus during which follicular development and female sexual receptivity occurs over the period of 1–9 days and concludes with ovulation (Spady *et al.*, 2007). A secondary increase or prolonged secretion of androgens reported for anovulatory females in this study may be related to multiple episodes of follicular development and re-occurrence of estrus (Spady *et al.*, 2007). Mating attempts, however, were unsuccessful at these later time periods suggesting that this time period may be too late for optimal breeding or conception, or that environmental conditions were not favourable for energetically demanding courtship behaviours.

It is difficult to discern whether the elevation of progestins post-breeding in ovulatory- non-parturient females indicates a lost pregnancy or pseudopregnancy. Tsubota *et al.* (1998) reported that unpaired female black bears produced a luteal phase of similar duration and concentration as the pregnant luteal phase. The observed increase in progestins for ovulatory non-parturient females in the present study, therefore, may be the result of the CL becoming functional during the correct time period, even though fertilization was unsuccessful. It is suspected that SB1147 had a pregnancy loss (aborted embryo) since the profile resembled that of a pregnancy 20–30 weeks after breeding. SB1041 was previously contracepted with both Depo-Provera and deslorelin, which may have led to a failure to conceive, or failure of the embryo to implant. Depo-Provera and other progestin contraceptives prevent pregnancy by altering uterine motility and endometrial receptivity, whereas deslorelin is a gonadotropin releasing hormone (GnRH) agonist and blocks the feedback mechanisms to the pituitary that lead to production of luteinizing and follicle stimulating hormones (Munson, 2006). Both contraceptives arrest ovarian follicular activity and could result in a failure of embryo implantation. Although they are thought to be eliminated over time, the effect of contraceptives on animal health and fertility have been of concern when used in felids (Munson, 2006). The health consequences of contraceptives use in ursids has not been documented and require further study.

Stirling *et al.* (2016) reported that the duration of wild polar bear breeding was brief (1 week of intense interaction, followed by only 5 days of active copulation), and once completed, animals separated and had no more interaction together. This mating period for wild animals was similar to the time reported for the interaction time of ovulatory female with males in this study (4–20 days). However, unlike the wild, captive animals can often have continued physical, visual or olfactory interaction with males beyond the brief period of mating. There are a number of factors that have been described to result in reproductive failure during embryonic diapause in mammals including poor nutrition and body condition, impaired health, or environmental stressors (Stringham, 1983; Palmer *et al.*, 1988; Eiler *et al.*, 1989; Atkinson and Ramsay, 1995). Moreover, unidentified pathology or subclinical conditions may also lead to lowered reproductive success. It is unknown whether any of these potential stressors impacted the suspected lost pregnancy described in this study; however, the above mentioned factors are all important points to consider in the reproductive management of polar bears in captivity.

Hormonal data from captive polar bears that better defines their reproductive physiology and breeding behaviours is translatable to wild population as more free-ranging females forego reproduction, abandon cubs, or cubs do not survive (Atwood *et al.*, 2016). Nutritional stress or disturbances of females during critical stages (e.g. embryonic diapause) of the reproductive cycle could lead to a greater number of pregnancy failures. Female polar bears have a limited number of times that they will enter estrus each year. Optimal time to breed may shift to earlier months of the year given the predicted disturbances that climate changes will have on the Arctic environment. Although a shift to an earlier estrus still corresponds to the natural gonadal cycle of males, changes in sea-ice conditions may physically place potential mates in distant habitats. Individuals have already been described to remain in different on-shore and sea-ice habitats at certain times of the year (Rode *et al.*, 2014; Atwood *et al.*, 2016). More fragmented and open sea ice may also prevent individuals from following scent trails of conspecifics (Owen *et al.*, 2015). Hormone biomarkers and reproductive characteristics should receive greater attention when assessing the individual and population level responses of polar bears to changes in their sea-ice habitat. An improved understanding of the reproductive physiology of captive animals lends insight into the potential that environmental stressors will have on the plasticity of polar bear reproductive ecology.
